# *The Organon* and Materia Medica Club of the Bay Cities of California

**Published:** 1898-07

**Authors:** Guy E. Manning

**Affiliations:** Secretary


### *THE ORGANON* AND MATERIA MEDICA CLUB OF THE BAY CITIES OF CALIFORNIA.

*The Organon* and Materia Medica Club was called to order January 7th, 1898, at 8.10 P. M., by the President. Present, Drs. J. M. and C. M. Selfridge, Augur, Holmgren, and Manning.

No other business presenting, the study of *The Organon* continued with Section 28, each paragraph being read by the President.

### DISCUSSION.

Section 28:

Dr. Selfridge—If it was not for some of the following paragraphs it would not be necessary to read this and Section 29, concerning the way remedies act.

Section 29:

Dr. Selfridge—Hahnemann thinks that the medicinal disease substitutes itself for the natural disease. That does not appear to be a very logical theory.

Dr. Augur—Nor to me either. If we were to judge of strength of disease by symptoms we would say the natural disease is stronger than the drug disease.

Dr. Selfridge—Hempel uses the word "antidotes," says "the drug antidotes the disease."

Dr. Augur—A better term, I should think.

Sections 30 and 31:

Dr. Selfridge—These paragraphs show that if a dose is big enough you feel the effect of the medicine. That is his idea, and that is our experience. A dose of physic in one man only large enough to act would tear another to pieces.

Sections 34 and 35:

Dr. Selfridge—No matter how strong the drug, it must be similar, or there would be no cure.

Sections 36 to 39:

Dr. Selfridge—All goes to prove that the drug disease can cure the natural disease if similar; if not similar, it can have no effect except, perhaps, to suspend natural disease for a time.

Section 42:

Dr. Selfridge—Hahnemann's theory in a few words is, that two similar diseases cannot occupy the system at the same time, the stronger taking the place of the weaker, but that two dissimilar diseases can and may occupy the system.

Section 45:

Dr. Manning—Then two drugs dissimilar in character can act in the same system?

Dr. Selfridge—Yes, if they are dissimilar, though the stronger disease will take the place of the weaker.

Section 49:

Dr. Selfridge—You see, Hahnemann realizes the fact that cow-pox is homœopathic to small-pox.

Dr. Augur—He proves it, too.

Section 51:

Dr. Selfridge—You will notice in potency he speaks of division to point of infinity; does not mention vital force.

Section 52:

Dr. Selfridge shows the entailment of a drug disease on top of natural disease. Does all this prove the forming of a drug disease in place of the natural disease? I do not consider his words aptly chosen in trying to prove the same. I consider Hempel's wording more satisfactory. Disease is evidently the result of changed vital force or molecular disturbance. Take a remedy. It starts the molecules revolving in right direction, if the right drug is chosen, and health is produced. But I do not know whether this theory is any more plain or satisfactory to you than the other.

Dr. Augur—Your explanation is, then, that disease is produced by disturbed molecular action, while the remedy produces vibratory action in right direction?

Dr. Selfridge—My idea is that disease disturbs molecular action so that the molecules do not act in normal direction—do not revolve in right manner.

Dr. Augur—You say, then, that the remedy restores normal action of molecules; then through similar action, not in dissimilar.

Dr. Selfridge—Oh, yes; the remedy must cause an action in the same plane, stopping the direction of the diseased molecules, restoring their correct action.

Dr. Augur—This is more plausible, I think.

Dr. Selfridge—Hahnemann's idea is by substitution—the drug absorbs the disease—is substituted for it. Not a plausible explanation in my mind. Or take the electrical idea of polarity, and suppose that in disease there is a change in the polarity of molecules; then health would be produced by a drug altering the position of these poles so that they would all point in same direction. One thing he does prove in these sections is that remedies which are not similar do not cure.

Sections 53, 54, 55:

Dr. Selfridge—That principle of attacking sound parts to relieve disease is practiced even now; it was employed in Hahnemann's day, and is now. I know that often the disease of the kidney will attack the liver and hide its former symptoms, but that does not stop the kidney trouble.

Section 56:

Dr. Selfridge—The palliative plan is the way to make money. In a case of appendicitis the palliator gives a hyperdermic injection, quiets the pain, gets the credit, while the case most probably goes on to suppuration and operation. The homœopath may take longer, but finds the remedy that will cure.

Adjourned. G. E. Manning,
*Secretary.*

